# Costs of vaginal delivery and Caesarean section at a tertiary level public hospital in Islamabad, Pakistan

**DOI:** 10.1186/1471-2393-10-2

**Published:** 2010-01-20

**Authors:** Attia Khan, Shakila Zaman

**Affiliations:** 1 Health services Academy, Chak Shahzad, Islamabad, Pakistan; 2 Institute of Public Health, Lahore, Pakistan

## Abstract

**Background:**

Public hospitals in developing countries, rather than the preventive and primary healthcare sectors, are the major consumers of healthcare resources. Imbalances in rational, equitable and efficient allocation of scarce resources lie in the scarcity of research & information on economic aspects of health care. The objective of this study was to determine the average cost of a spontaneous vaginal delivery and Caesarean section in a tertiary level government hospital in Islamabad, Pakistan and to estimate the out of pocket expenditures to households using these services.

**Methods:**

This hospital based cost accounting cross sectional study determines the average cost of vaginal delivery and Caesarean section from two perspectives, the patient's and the hospital. From the patient's perspective direct and indirect expenditures of 133 post-partum mothers (65 delivered by Caesarean section & 68 by spontaneous vaginal delivery) admitted in the maternity general ward were determined. From the hospital perspective the step down methodology was adopted, capital and recurrent costs were determined from inputs and cost centers.

**Results:**

The average cost for a spontaneous vaginal delivery from the hospital's side was 40 US$ (2688 rupees) and from the patient's perspective was 79 US$ (5278 rupees). The average cost for a Caesarean section from the hospital side was 162 US$ (10868 rupees) and 204 US$ (13678 rupees) from the patient's side. Average monthly household income was 141 ± 87 US$ for spontaneous vaginal delivery and 168 ± 97 US$ for Caesarean section. Three fourth (74%) of households had a monthly income of less than 149 US$ (10000 rupees).

**Conclusion:**

The apparently "free" maternity care at government hospitals involves substantial hidden and unpredicted costs. The anticipated fear of these unpredicted costs may be major factor for many poor households to seek cheaper alternate maternity healthcare.

## Background

Pregnancy a normal, healthy state which most women aspire to at some point in their lives, carries with it serious risks of death and disability [1]. Over half a million young women die every year as a result of complications arising from pregnancy and childbirth [2], most of these deaths occur in the developing world.

Reduction in maternal mortality rates as observed in most high-income countries was achieved by providing access of pregnant women to skilled care during pregnancy and childbirth and to the guaranteed provision of safe interventions such as assisted vaginal delivery and Caesarean section [3].

Pakistan with a maternal mortality ratio at 297/100,000 live births [4] has seen a slow rise in the proportion of pregnant women receiving prenatal care from a skilled health professional increasing from 43% in 2001/02 [5] to 50% in 2004/05 [6] and lately to 61% in 2006-2007 (78% urban and 54% rural women) [7]. But unfortunately 64% of pregnant women (74% rural and 43% urban) in Pakistan still deliver at home [4].

In developing countries public hospitals, rather than the preventive and primary healthcare sectors, are the major consumers of healthcare resources [8]. Local health planners have inadequate knowledge of the costs of the healthcare services such as costs of running the in-patient hospital services they render [9,10]. Through technical and allocation efficiency and rational priority settings guided by a sound knowledge of the costs, scarce resources can be used efficiently. A report by Green and Ali [11] discusses two costing studies undertaken in Balochistan (a province in Pakistan) by Ali et al and Ali and Naeem. According to the report, information on costs and expenditures of different health facilities and health services in Pakistan needed to base rational decisions on is almost non existent. The application of these research studies was to develop a decentralized budget.

In view of the importance of cost exercises in healthcare management many developing countries, undertook costing studies. For example Vietnam [12] and Argentina [13] used costing studies to measure efficiency and wise allocation of public funds. Costing studies are also useful for countries undergoing health reforms such as decentralization [14] and hospital autonomy. In India [15] and Kenya [16] cost analysis was an important element in decisions on setting levels of user fees. WHO collated data [17] on unit costs from many hospitals and countries, many countries had absolutely no information on unit costs and studies to determine the costs of maternal health services in developing countries [11,18] were scarce.

Public health environment in Pakistan challenges policy makers to increase the quantity and quality of health services, but the resources available to improve these services are by and large insufficient. The healthcare budget (0.5% of government expenditure) is far below the 5% recommended for developing countries by WHO and the annual incremental budget allocates resources that have little relation with the healthcare needs of the population or the requirements of the facility to function efficiently.

The objective of this study is to estimate the average cost of a spontaneous vaginal delivery and a Caesarean section delivery at a tertiary level government hospital. This will provide an insight to hidden and real costs involved in provision of maternal health services by the government and to the households availing these services. Information gained from this study can be used to identify areas where costs could be reduced and where output or productivity could be increased. It may be used as a resource tool for financial management in hospitals and for suggesting measures (example health insurance and premiums) in making maternal healthcare more affordable.

## Methods

### Study design

The study was a hospital based cost accounting cross sectional study estimating the average cost of spontaneous vaginal delivery (SVD) and Caesarean section (CS) from the provider (hospital) perspective and the user (patient) perspective.

Duration of the study was from 1^st ^April 2008 to 30th June 2008

### Study area

The study was carried out at a large government Maternity and Child Hospital (MCH) located in Islamabad, Pakistan. The hospital, a teaching and general referral hospital provides maternity services to women of the twin cities of Islamabad and Rawalpindi and also the districts of Attock and Nowshera (total population of over 6 million in 1998).

### Patient perspective

#### Sampling technique and size

To estimate the out of pocket expenditure 133 postpartum mothers (68 delivered vaginally and 65 delivered by Caesarean section) all Pakistani Asian in origin were interviewed at the hospital between 10^th ^April and 10^th ^May 2008. Convenience sampling technique was used for selection of interview candidates from the vast variety of obstetric cases in the general ward as there was a high possibility of not acquiring the required sample within this short duration of the study. All new admissions and all cases to be discharged were identified after major ward rounds (two to three in 24 hours). From these all post partum mothers fulfilling the selection criteria (including uncomplicated SVD patients with very short hospital stay) were selected for interview. Patients with a CS or a complicated SVD were visited daily and all costs and expenditures till their discharge from the ward were noted.

To calculate the sample size the statistical formula to demonstrate a significant difference between two groups by comparing proportions was used [18] at a two sided α value of 0.05 (1.96) and confidence interval of 95% allowing the study power to be 90%. Caesarean section rate varies from 0-40% globally [19] and it was taken as 23% (CS rate was 23.2% in the U.S.A) [20]. Refusal rate very was low at 0.75%.

#### Sample recruitment

Inclusion criteria

• All post partum mothers delivering at term (36-42 weeks of gestation) either by spontaneous vaginal delivery or Caesarean section

Excluded were post partum mothers

• Admitted in private ward/rooms

• Delivered by instrumental (forceps) vaginal delivery

• Exempted from hospital dues

• Not delivered at the hospital but admitted for post partum complications

The majority of women admitted in the private maternity ward/rooms belonged to the upper socioeconomic class, including the wives of government officers; therefore they were not included in the study. A small proportion of vaginal births/deliveries at the hospital were forceps vaginal deliveries which were excluded from the study as their process and outcome varied from that of spontaneous vaginal delivery.

#### Risk profile of the study groups

There is evidence that a number of maternal, neonatal, clinical, socio-demographic, and economic risk factors are associated with Caesarean section delivery. We measured some of these risk factors in the two study groups (CS and SVD). The variables measured were demographic risk factors such as maternal age, education, parity; clinical risk factors such as maternal co-morbidities (diabetes, hypertension, hepatitis B, hepatitis C and anemia); neonatal risk factors such as sex of baby, birth weight of baby and economic risk factors such as socioeconomic status of patient (total monthly income).

#### Cost factors

To estimate the total cost of a delivery (CS or SVD) to a household information on direct and indirect costs such as expenditure on food, transport, drugs, tests, blood transfusion, informal caregiver's time cost (opportunity cost), hospital dues and informal payments (tips and bribes) was also obtained through interviews.

A pre-tested semi-structured questionnaire was used to interview post partum mothers in the ward. As majority of mothers were no able to provide sufficient details and lacked accuracy on costs and expenditures the help of their husband or a close relative was sought. All relevant expenses till discharge from the hospital were accounted for. Information on expenditures was usually supported with payment receipts provided by husbands and relatives.

Opportunity cost measures how much is "given up" in terms of real cost by carrying out an activity [21]. A more specific term "the informal caregiver's time cost" used by Islam MK in a WHO publication [22] relates to the cost of time spent in informal care (not paid) by friends and family. Opportunity cost was determined after classifying the work status of the care givers and the patient as salaried work, unemployed or housework. Housework was given a monetary value of 45 US$ (3000 rupees per month) the minimum permissible salary in Pakistan. Unemployment was not given any monetary value. Intangible costs [23] which reflect the patient's level of pain and suffering and the limitations it imposes on the quality of life are difficult to measure and were not measured in this study.

### Provider (hospital) perspective

Three types of methodologies can be employed to calculate unit costs, the activity based approach, the top-down approach and the bottom-up approach [24]. To estimate the provider costs the top-down approach was employed in this study. A tool was developed to gather data on capital and recurrent costs.

The top down approach begins at the top classifying costs as capital and recurrent costs. Capital cost is expenditure on goods which last longer than one year, such as investment in equipment and infrastructure [25]. Recurrent costs or operating costs are costs associated with the operation or maintenance of facilities or assets.

The main inputs of capital costs were land, building, equipment and vehicles. Data on capital costs was obtained from hospital records and by direct interview of personnel from various hospital departments of finance, administration, engineering works and transportation. The useful life of buildings (70 years), equipments and vehicles (10 years) as documented in the hospital records as per government rule was utilized to determine depreciation with time of capital inputs. The average capital cost per SVD and CS was then determined by; cost per bed per day × average length of stay.

Recurrent costs were categorized into three levels of cost centers. (Refer to table 1)

**Table 1:** Break up of recurrent costs into cost centers

Overhead cost centers *Shared services*	Intermediate cost centers *Directly rendered services*	Final cost center *Personnel services*
Administrative costs	Blood bank	Officers salary
Transport & travel costs	Laboratory	Staff salary
Repair & maintenance	Diagnostics	
Linen, laundry & housekeeping		
Utilities (gas, electricity, water)		
Communication (post, telephone & telegraph		
Occupancy costs (rent & taxes)		
Drugs & supplies		
Recurrent training		
Transfer payments		

• Unit costs of overhead cost centers;*shared services *such as drugs, laundry, administrative costs etc

• Unit costs of intermediate cost centers; *directly rendered services *such as blood bank, laboratory and diagnostics

• Unit costs of final cost centers; *personnel services *such as staff salaries

Information on overhead and final cost centers was obtained from the annual budget and expenditure report from the hospital's account section. Services that came under the intermediate cost centers such as blood bank, and laboratory were not covered by the MCH hospital budget. These services were shared with other departments of the hospital. The information on these services was obtained from the relevant departments. The various activities of doctors and nurses such as time spent in patient care, administrative work and personal time could not be analyzed due to un-willingness of staff to be monitored and the non availability of individualized salary information (Information on staff salaries was accessible only as bulk payments)

### Data analysis

The cost data from both provider and patient perspective was entered separately on Microsoft Excel to obtain total costs, average, minimum, maximum and percentages. Non cost data from both study groups (SVD and CS) was analyzed on SPSS version 15. The variables analyzed were mother's age as discrete numbers and in groups (18-25 years, 26-30, 31-35, 36-40, 41-45), baby's weight (1-2.5 kg, 2.6-3, 3.1-3.5, 3.6-4), household income, length of stay, number of living children, total cost of delivery, educational level of mother (no formal education, primary, secondary, college and university), co-morbidities (disease free, diabetes, hypertension, anemia, hepatitis C and others) and sex of baby (male vs. female). The number of cases with missing values was very small (1.5%) as compared to the sample size therefore cases were dropped from analysis on SPSS by list-wise deletion.

Frequencies and descriptive analysis was performed for different variables. Both simple linear regression (SLR) and multiple linear regressions (MLR) were conducted using total cost of delivery as dependent variable and length of stay, household income, baby's weight, age of mother and number of living children as independent variables.

Non parametric bootstrapping technique was applied to generate confidence intervals for cost data. We used the chi-square test to examine the existence of an association between mode of delivery (vaginal vs. Caesarean) and educational level of mother, co-morbidities and sex of baby.

### Ethical considerations

The study was approved by the ethical committee of Health Services Academy and the government hospital where the study was conducted. Informed verbal consent was taken from the mothers.

## Results

### Overview

The average cost from the hospital's side for a spontaneous vaginal delivery was 40 US$ *(2688 rupees) *and a Caesarean section was 162 US$ *(10868 rupees) *(Refer to table 2). The average cost from the patient's side of a spontaneous vaginal delivery was 79 US$ *(5278 rupees) *and a Caesarean section was 204 US$ *(13678 rupees)*. One US dollar [26] is equivalent to 67 Pakistani rupees (May 2008).

**Table 2:** Capital and recurrent costs

		Costs converted to US$			Input cost as % of	
		
Cost category	Input cost	Annual Hospital	Per SVD	Per CS	Capital/recurr	Total Cost
**Capital**	**Medical equipment**	131411	3	13	48.4	8
	**Building & commodities**	122939	3	119	45	7.4
	**Bank & consultancy**	15308	0.4	1.5	5.7	0.057
	**Vehicles**	1070	0.03	0.1	0.4	0.06
	**Land**	110	0.003	0.01	0.04	0.0007

**Total capital**		**270838**	**6.6**	**26.4**	**100**	**16.8**

**Recurrent**	**Personnel**	574845	14	56	41	35
	**Transfer payments**	83261	206	8	6	5
	**Utilities**	270561	7	27	19.5	16
	**Drugs**	135284	3	13	9.7	8.2
	**Occupancy**	103162	2.5	10	7.4	6.3
	**Administration**	51162	1.3	5	3.7	3
	**Repair & maintenance**	35051	0.9	3.4	2.5	2.1
	**Blood bank**	180187	0.6	3.4	2.5	1.5-2.0
	**Plant purchases**	30746	0.7	3	2.1	1.9
	**Communication**	26442	0.6	2.6	1.8	1.6
	**Transport**	14447	0.4	1.4	1	0.9
	**Laboratory**	223881	0.3	1.5	1	0.6-0.9
	**Housekeeping etc**	11930	0.3	1.2	0.9	0.7
	**Training**	5780	0.1	0.6	0.4	0.3
**Total recurrent**		**1341165**	**34**	**136**	**100**	**83.2**

**TOTAL**		**1612002**	**40**	**162**		**100**

### Average cost of delivery; the provider perspective

From January 2007 to January 2008, a total of 10001 births (all modes of deliveries) took place at the hospital. Of the total indoor admissions 80.6% were related to deliveries. During the same year the hospital Caesarean section rate was 25% (remained quite constant throughout the year) and bed occupancy rate was 90%. According to hospital statistics average length of stay for a spontaneous vaginal delivery was one day while that of a Caesarean section was four days.

In our study recurrent costs comprised 84% of total costs while capital costs comprised 16% of total cost for both modes of delivery (refer to table 2). The two major inputs of capital costs were medical equipments and building contributing 48% and 45% respectively.

Personnel cost was the largest cost components of total cost and recurrent cost, contributing 35% to the former and 41% to the later. Transfer payments were mainly scholarships to house-officers and postgraduate trainees. On including them in personnel cost, staff salaries costs reached a 40% of total cost. Expenditure on drugs & supplies was 8.2% of total costs where as utilities (natural gas, electricity and water) took up 16% of total cost.

### Average cost of delivery; the patient perspective

**Figure 1:**
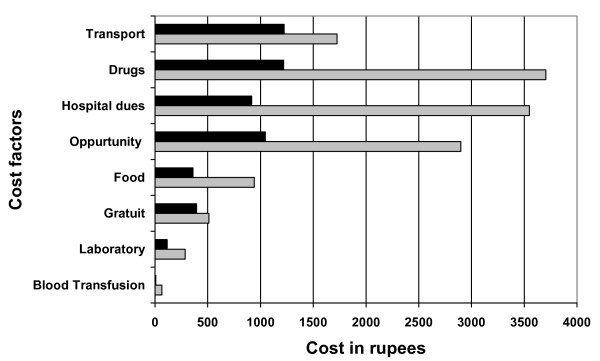
**"Black box" represents cost factors for spontaneous vaginal delivery "Grey box" represents cost factors for Caesarean section delivery**.

The two major cost components of spontaneous vaginal delivery were transportation and drugs, each contributing 23% to total cost, whereas drugs (27%) and hospital fees (26%) were the largest cost components of Caesarean section. Refer to figure 1.

### Profile of post partum mother

Major proportion of mothers (43% of SVD and 35% of CS) was educated up to secondary school level. Illiteracy on the other hand was not as high as expected, though it was comparatively higher in the SVD group at 23% vs.15% in CS group. Majority of mothers from the SVD group 85.3% and 60% of CS group had no associated disease. Hypertension and anemia were the most frequently observed co morbidity in both groups. Hypertension (mostly pregnancy induced) was three times and anemia was eight times more prevalent in the CS group as compared to the SVD group. Refer to table 3.

**Table 3:** Profile of study groups: SVD and CS

	SVD		CS	
**Character**	**Mean ± SD**	**Range**	**Mean ± SD**	**Range**

Maternal age in years	25 ± 6	18-44	27 ± 6	17-39
No. of alive children	2 ± 2	1-10	2 ± 2	1-12
Weight of newborn -Kg	2.8 ± 0.5	1.2-4.4	2.7 ± 0.7	1.1-4.2
Household income-US$	141 ± 87	45-447	168 ± 97	45-418
Length of stay -days	1.4 ± 0.9	1-5	4 ± 1.3	2-8
Total costs borne-US$	79 ± 37	30 ± 193	204 ± 60	70-482

**Education level**	**Frequency**	**Percent**	**Frequency**	**Percent**
No formal education	16	23.5	10	15.4
Primary	12	17.6	8	12.3
Secondary	24	35.3	28	43
College	7	10.3	11	17
University	9	9	8	12.3

**Co-morbidities**				
Disease free	58	85.3	40	61.5
Diabetes	2	2.9	2	3.1
Hypertension	5	7.4	14	21.5
Hepatitis C	2	2.9	0	1.5
Anemia	1	1.5	8	12.5

**Household income** **Pak Rupees US$**				
3000-6000 (45-90)	23	34	21	32
6000-10000 (90-149)	27	40	14	22
10000-15000 (149-223)	9	13	15	23
15000-20000 (223-298)	4	6	7	11
20000-30000 (298-447)	5	7	8	12

Pearson chi-square analysis showed a significant association of maternal age, co morbidity and number of living children (parity) with mode of delivery (CS vs. SVD). Strongest association was seen with length of stay (LOS) with P < 0.0001 which was quite obvious.

Simple linear regression analysis revealed that neither baby's weight, nor maternal age or parity is a significant variable when assessed for total charges. These conclusions did not change after other factors were controlled in SLR and stepwise MLR. The final MLR model was significant (*P *< 0.000) and R^2 ^was 0.681. Length of stay (LOS) and household income remained significantly associated with the final model. The results indicate that the bulk of the variation associated with the total cost of delivery is explained by length of stay in the ward in this model and household income.

## Discussion

The average combined patient and provider cost on a SVD is 119 US$ (7966 rupees), 66% of this cost is borne by the patient, while the combined cost on a CS is 366 US$ (24546 rupees), 56% of this cost is borne by the patient.

### Affordability

One third (33%) of households from both groups (SVD & CS) earned less than 90 US$ (6000 rupees) per month. Households earning less than 149 US$ (10000 rupees) per month consisted 74% of SVD and 54% of CS. With regard to household income, delivery costs are far beyond the limits of many poor families. To many households the fear of unforeseen expenditures, high direct and indirect payments for a facility based delivery may pose to be a barrier to the use of maternity services. A similar study by Nahar and Costello [27] estimated that 79% of households in Bangladesh did not have enough money to pay for delivery and they had to borrow from friends and relatives.

### Imbalance in resource allocation

The major burden of drugs and transport was borne by households for a spontaneous vaginal delivery where as provision of drugs & supplies and paying the hospital dues for a Caesarean section was a challenge for poor households. Kowaleski's study [28] on maternity services in rural Tanzania similarly reports admission charges, drugs and travel as major costs for households.

Recurrent costs were the major costs (84%) from the provider/hospital side. Of the total cost (capital and recurrent) 40% was spent on staff salaries and a meager amount of 8.2% was spent on purchase of drugs & supplies (an imbalance in resource allocation is evident). Studies from India [8], Zimbabwe [14] (Plaetse) and Argentina [13] (Borghi) gave variable results, 54%, 61.48% and 88-90% of total cost was spent on staff salaries.

According to a World Bank [29] report the cost of a normal vaginal delivery at a hospital in poor countries of Africa and Latin America ranges from US$ 10-35 and a Caesarean or a complicated vaginal delivery can cost from US$ 50-100. The average cost of a SVD (40 US$ or 2688 rupees) and a CS (162 US$ or 10868 rupees) in our study was substantially higher than other poor countries of Africa and Latin America but more comparable to results from regional studies from Bangladesh [27] (year 1995, SVD: US$ 31.9 and CS: 123 US$ from patient perspective) and India [15] (SVD; 8215 rupees and CS; 7012 rupees).

Higher costs in our study could be accounted for as a regional variation or due to rapidly rising cost of maternity healthcare over the years.

### Risk profiles

Maternal age, higher education and co morbidity were directly associated with Caesarean section in this study. In a study from Tabriz [30] higher level of education, maternal age and socioeconomic status were associated with Caesarean section. A cohort study from England [31] revealed that increasing maternal age, diabetes mellitus, neonatal birth weight and head circumference were associated with an increased risk of a CS while increasing parity was associated with a decreased risk of CS. A cross sectional study from Beirut [32] identified gestational age, multiple gestations, number of previous deliveries, time and date of delivery and site of antenatal care (private vs. public) to be related to increase in CS rate.

### Study limitations

Precise costing of time for activities (patient care, administrative work and personal time) of health care workers such as doctors and nurses in the hospital could not be performed due to unwillingness of staff to be monitored and due to non-availability of individualized salary data. There was some limitation of quality of the cost data on services that were shared amongst patients in MCH and patients from other departments of the main hospital. These shared services included the blood bank and laboratory (pathology, biochemistry etc). Record of budget and expenditure on these services was available but accurate data on number of maternity patients taking the tests and the number and kinds of tests taken by each maternity patient was not documented in a manner to fulfill the requirements of the study.

Another limitation of our study was the inability to interview a small number of patients arriving during the night for an uncomplicated vaginal delivery and leaving the hospital before the morning ward round (leave against medical advice). On examining such patient's files the treatment provided to them was not any different from patients that stayed a few hours longer to be properly discharged after the morning ward round.

The costs of delivery in our study are representative of costs at tertiary level public hospitals but may not be representative of costs in rural or semi-urban settings, at primary & secondary healthcare level nor is it representative of deliveries in the private sector.

There is a possibility that costs of Caesarean section may have been underestimated as some aspects such as peri-natal, natal and post natal risks and outcome, increased risk of placenta preavia and elective Caesarean section have not been covered. A study on a larger scale with a wider time zone is required to cover all aspects of Caesarean section and its associated costs.

## Conclusion

Despite of the 10000 annual births, the 90% bed occupancy and the high bed turnover, costs of vaginal delivery and Caesarean section in a tertiary level public hospital in Islamabad city are substantially higher than other studies in other developing countries. Regardless of maternity healthcare being subsidized by the government, the costs of a delivery whether be it a vaginal delivery or a Caesarean section is far beyond the limits of three fourth of households in Pakistan. Effective methods of health insurance tailored to the local needs should be introduced to make maternal healthcare more affordable to the poor and average households.

## Abbreviations

CS: Caesarian section; SPSS: Statistical Program for Social Sciences; SVD: Spontaneous vaginal delivery; U.S.A: United States of America; W.H.O: World Health Organization; SLR: Simple linear regression; MLR: Multiple linear regression; LOS: Length of stay; MCH: Maternal and Child Health

## Competing interests

The authors declare that they have no competing interests.

## Authors' contributions

AK proposed the study, contributed to acquisition, analyses and interpretation of data and drafted the manuscript. SZ supervised the study design, statistical analysis and critical review of important intellectual content of the manuscript. All authors read and approved the final manuscript.

## Pre-publication history

The pre-publication history for this paper can be accessed here:

http://www.biomedcentral.com/1471-2393/10/2/prepub
